# Medium-term biological variation of sodium, potassium and chloride in whole blood in cohorts of healthy individuals and hemodialysis patients

**DOI:** 10.1515/almed-2026-0012

**Published:** 2026-07-01

**Authors:** Berta Sufrate-Vergara, Isabel Moreno-Parro, Pilar Fernández-Calle, Aasne K. Aarsand, Sverre Sandberg, Anna Carobene, Roberto Mora Corcovado, Daniel Prieto Arribas, Antonio Buño Soto, Paloma Oliver, Blanca Beumer Prieto, José Manuel Hidalgo, Ana Cristina Mendoza, Sara Julia Molina, Nuria Alert, Ana Monreal, Jorge Díaz-Garzón Marco

**Affiliations:** Laboratory Medicine Department, La Paz University Hospital, Madrid, Spain; Health Research Institute of La Paz University Hospital (IdiPAZ), Madrid, Spain; Norwegian Organization for Quality Improvement of Laboratory Examinations (Noklus), Haraldsplass Deaconess Hospital, Bergen, Norway; Laboratory Medicine Department, IRCCS Ospedale San Raffaele, Milano, Italy; Nephrology Department, La Paz University Hospital, Madrid, Spain

**Keywords:** biological variation, electrolytes, hemodialysis, whole blood

## Abstract

**Objectives:**

Biological variation (BV) refers to the fluctuation of the concentration of a measurand around its homeostatic set-point. While BV has been extensively studied in healthy populations, robust data for patients with chronic diseases, particularly using whole blood measurements, remain scarce. The aim of this study was to obtain BV estimates for electrolytes in whole blood for a cohort of healthy individuals and one of hemodialysis patients (HD) and compare them with each other.

**Methods:**

A prospective BV study was conducted in 18 stable HD patients and 26 healthy individuals. Whole blood potassium, sodium and chloride concentrations were measured weekly for 12 consecutive weeks using a direct potentiometry blood gas analyser. Preanalytical conditions were standardised. BV estimates were delivered using both CV-ANOVA and Bayesian methods. Population homogeneity was assessed using the Harris–Brown heterogeneity ratio, and the index of individuality was calculated for each measurand.

**Results:**

Significant differences in BV estimates were observed between groups. BV estimates were higher in HD patients for most measurands. Within the healthy group, whole blood within-subject (CV_I_) estimates were higher than those reported for serum/plasma in the literature. Heterogeneity analyses indicated non-homogeneous CV_I_ distributions for sodium and potassium in HD patients.

**Conclusions:**

This study provides the first BIVAC-compliant BV estimates for electrolytes measured in whole blood. These results highlight significant disease-related differences in BV and support the need for disease- and matrix-specific reference tools to enable personalised interpretation of electrolyte results in hemodialysis monitoring.

## Introduction

Biological variation (BV) is defined as the fluctuation of the concentration of a measurand around its homeostatic set-point (HSP) [1], 2]. BV is divided into two components depending on whether it refers to the variation within the same individual (within-subject BV, CV_I_) or the variation between the HSPs of different individuals in a population (between-subject BV, CV_G_).

Traditionally, laboratory results are interpreted by comparison either to population-based reference intervals (RIs) or to clinical decision limits (CDLs), with the latter established through outcome-based studies [3]. However, the use of RIs has certain limitations, such as that serial results from a single individual do not necessarily fall within the interval defined for the reference population, particularly for measurands with tight homeostatic regulation. Furthermore, the criteria to establish these CDLs are, in many cases, arbitrary and depends on the study design, population and different outcomes.

Linked to the notion of BV, two useful statistical concepts have been developed for interpretation of laboratory results: the reference change value (RCV) [4] and personalized reference intervals (prRIs) [2]. RCV allows assessment of whether the difference between an individual’s repeated results is larger than what can be explained by biological variation (BV) and analytical noise (analytical variation, CV_A_). This can assist clinicians to judge whether an observed change in the concentration of the measurand under study is likely related to an alteration in the patient’s condition. prRIs represent a novel concept in which information about the HSP is combined with BV data are used to calculate a personalized RI, which will typically be narrower and more fitted to the individual than traditional population-based RIs [2].

To obtain robust, high-quality BV data and to apply them appropriately to result interpretation, it is essential that studies are conducted using rigorous methodological design and appropriate data handling, as recommended by the Committee on Biological Variation (C-BV) of the European Federation of Clinical Chemistry and Laboratory Medicine (EFLM) [5]. In collaboration with the Technical Committee on the Biological Variation Database (TC-BVD), the groups developed a critical appraisal checklist for the methodological evaluation of BV studies (BIVAC) [6]. Following a literature review and meta-analysis of the available studies, the TC-BVD published the resulting data in the EFLM Biological Variation Database [7], which is continuously updated.

BV has been extensively studied for many measurands in healthy individuals. However, there is generally a lack of robust data for other states of health and stable diseases. There are data that indicate that certain conditions – such as physical exercise [8], [9], [10], pregnancy [11], 12], or chronic diseases [13], [14], [15], [16], [17], [18] – may influence the fluctuation of specific measurands around their HSP, making the CV_I_ different from that observed in the general population. Considering that pathophysiological characteristics of different diseases are different, it is possible that BV will also differ for the same measurands when assessed for diverse pathological conditions. If using BV data derived from the general healthy population for the assessment of an unhealthy patient, it is important to know if the BV data are relevant for that patient population. If this is not the case, RCV and prRIs should be calculated using BV estimates derived from a representative patient cohort with the disease of interest, when in a stable state. There are published studies on BV in chronic diseases (diabetes [17], 19], 20], chronic kidney disease [15], 16], [21], [22], [23], cancer [18], heart failure [13], 14], 24]), but the parameters studied are not always the ones most closely related to the disease or the methodology does not always follow current recommendations [5], 6] and, therefore, the estimates may not be correct. Furthermore, for many measurands important for monitoring patients with chronic diseases, BV studies are lacking.

Chronic kidney disease (CKD) is highly prevalent, affecting about 15 % of the Spanish population [25] and is associated with alterations in multiple biomarkers (i.e. electrolytes, creatinine, hemoglobin…). Many CKD patients remain in a stable disease state for many years. In such a setting, the application of a more personalised approach for early detection of changes is likely to be useful. Also, electrolyte disturbances are common in CKD patients. Here the availability of rapid point-of-care blood gas analysers measuring electrolytes in whole blood allows for timely assessment of these markers at bedside. This facilitates early detection of electrolyte imbalances and prompt clinical decision-making, follow-up and treatment adjustments.

In light of the considerations outlined above and the limited availability of BV estimates for electrolytes measured in whole blood, a BV study was conducted in healthy individuals and in patients undergoing hemodialysis, with the aim of generating robust BV estimates for both populations and enabling direct comparison between them.

## Materials and methods

### Study population

The study population comprised two groups of participants (44 in total) recruited at a tertiary hospital in Madrid, Spain as part of the same research project:

– Hemodialysis patients (HD): 18 adult patients (13 males and 5 females) with CKD undergoing replacement therapy with hemodialysis in the Nephrology Department. The median age was 78 years (range: 50–94). Patients had to have been attending hemodialysis three times a week for at least three months at the time of inclusion and be in a stable disease phase according to their doctor’s evaluation. Exclusion criteria were recent cancer history (previous six months), poor hemodynamic tolerance to dialysis and poor treatment adherence.
– Healthy individuals (HI): 26 healthy volunteers (6 males and 20 females) recruited mainly among hospital workers. The median age was 35 years (range: 25–62). Inclusion criteria were being over 18 years old, not having recent hospitalization history, not suffering from any chronic disease and not being pregnant.

The host institution Research Ethical Committee approved the study, and all the participants signed Informed Consent.

### Sample collection

Whole blood samples were collected in syringes containing balanced heparin weekly for 12 consecutive weeks between 8 and 10 am. In the HI group, samples were always taken by one of the same team of nurses at the hospital’s Sample Collection Ward. Samples from the HD group were collected at the unit where they received treatment. Blood collection was performed by their nursing team and samples were always taken before starting dialysis on the middle day of the three-day treatment (i.e. if the patient underwent dialysis on Mondays, Wednesdays and Fridays, samples were collected on Wednesdays).

The preanalytical phase was standardized [6], 26] to minimize variability and samples were immediately sent by pneumatic tube to the laboratory after collection for processing within 30 min.

### Sample analysis

Whole blood concentrations of potassium (K^+^), sodium (Na^+^) and chloride (Cl^−^) were determined. The analyser used was the ABL90 from Radiometer (Denmark), with a direct potentiometry method. The instrument was in the Laboratory Medicine Department, which is accredited to ISO 15189 standards.

All measurements were performed in duplicate on the same analyser to avoid possible bias due to equipment changes. Due to stability requirements, it was not possible to analyse all participant’s samples within a single analytical batch; instead, samples were processed as participants attended their scheduled visits.

### Data analysis

The distribution of concentrations for each sample from each participant was evaluated using the Shapiro–Wilk test and medians together with interquartile ranges (IQR) were calculated for all measurands. Differences between groups (HD and HI) were assessed using the Mann–Whitney U test. For it to be considered statistically significant, p needed to be below 0.05.

Trend analysis was performed to assess whether participants were in a steady state. This was done by fitting a regression line for each subject; a trend was considered statistically significant when the 95 % confidence interval of the slope did not include zero, in which case, subject-specific analyses were conducted and affected individuals were excluded.

BV components were estimated using two different statistical approaches: an ANOVA-based model and a Bayesian model.

The CV-ANOVA approach has proven to be robust for estimating CV_A_, CV_I_ and CV_G_ in nested random-effects designs. Prior to applying CV-ANOVA, outlier detection across replicates and samples from the same individual were assessed. The method applied was Cochran C, assuring homogeneity of variances. Outliers between individuals were assessed by Reed’s criterion. All outliers identified were excluded from the final dataset. A logarithmic transformation was applied to obtain CV_G_^4^.

Implementation of the Bayesian model requires prior information (priors or hyperparameters) on BV estimates for each one of the measurands under study. Priors were obtained from the EFLM Biological Variation Database [7] and down-weighted in the model (25 % SD). Priors for CV_A_ were established based on internal quality control data analysed concurrently with study samples. The Bayesian models crudely estimates the individual, within-person BV (CV_P(I)_) with their predicted distributions. Harris–Brown heterogeneity ratio (HBR) was calculated as 100 % × (σ[CV_P(I)_]/μ[CV_P(I)_]) [27]. HBR is a measure of variability in relation to the mean in the population and is expected to be below 100 %/sqrt(2s) for a homogeneous population with s being the average number of samples from each individual. The model assumes Student’s t distributions to enhance robustness against extreme values, thus exclusion of outliers is not required. The index of individuality (II) was calculated as the CV_I_ to CV_G_ relation for both the ANOVA and Bayesian approaches [28].

All statistical analyses were conducted using RStudio (v.2023.09.01) with the Rstan (v.4.0.2), lme4 (v.1.1-35.5), and Tidyverse (v.2.0.0) packages. The main codes are publicly available in previously published studies [29], 30].

## Results

**Figure 1:**
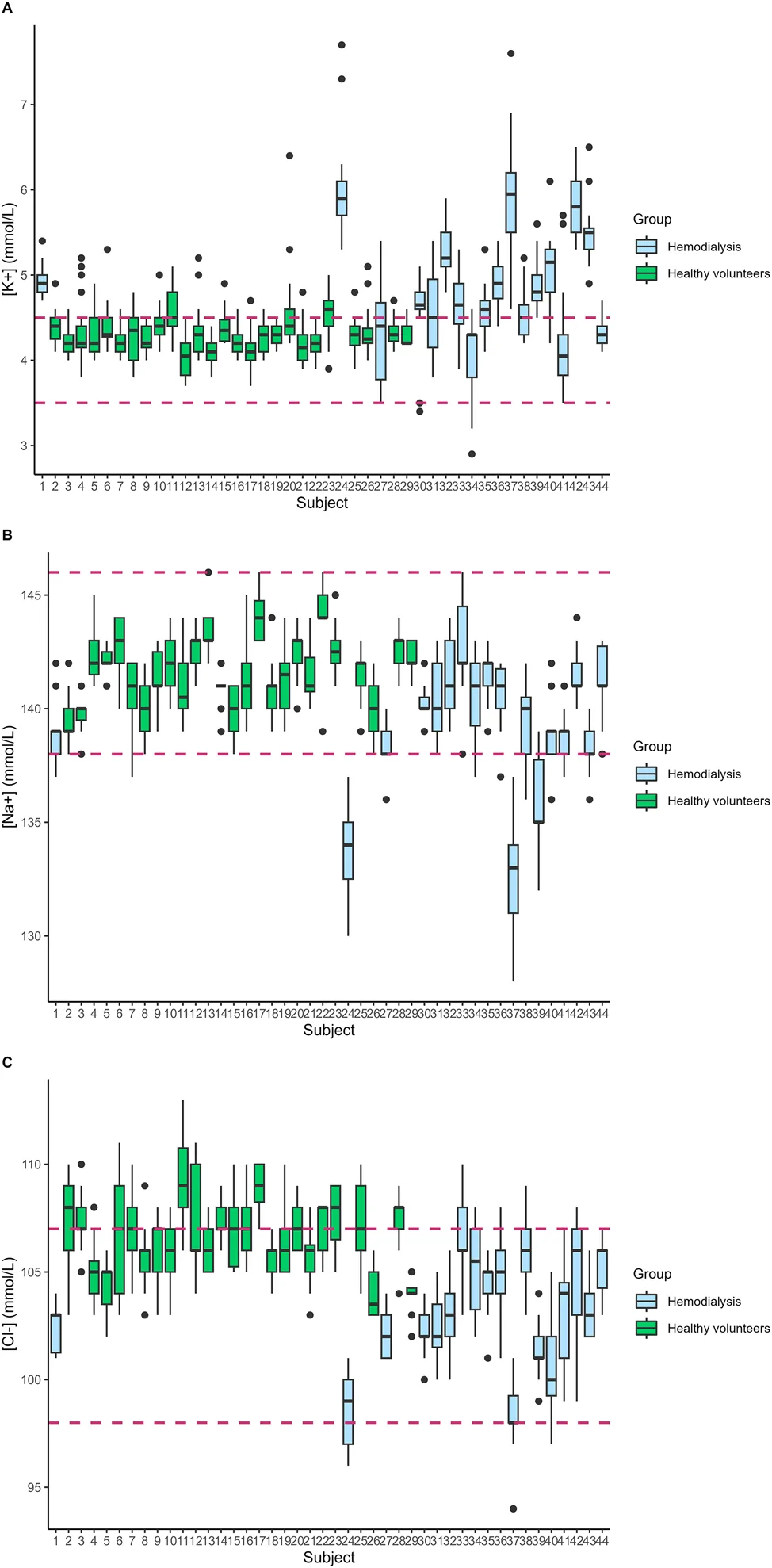
Electrolytes’ concentration all over the time (weekly sampling) for the individuals included (A) K^+^, (B) Na^+^, (C) Cl^−^. Each boxplot represents a different participant. Different colours indicate the subgroup: hemodialysis patients in blue and healthy individuals in green. The dashed burgundy lines represent the reference intervals established in our laboratory: K^+^ (3.5–4.5 mmol/L), Na^+^ (138–146 mmol/L), Cl^−^ (98–107 mmol/L). Reference intervals are defined for plasma samples.

Raw data about electrolytes’ concentration for the individuals included in the study is presented in Figure 1. The mean number of samples collected per subject was 10.9 for HD and 10.5 for HI.

The data of none of the three analytes followed a normal distribution. Median concentrations are shown in Table 1. Statistically significant differences were observed between the two cohorts for all three electrolytes.

**Table 1:** Median concentrations with interquartile range (IQR) for electrolytes in healthy individuals and patients on hemodialysis.

Measurand	Units	Group	Median	IQR	p-Value
K^+^	mmol/L	Hemodialysis	4.8	0.9	**<0.001**
		Healthy individuals	4.3	0.2	
Na^+^	mmol/L	Hemodialysis	139	3	**<0.001**
		Healthy individuals	142	3	
Cl^−^	mmol/L	Hemodialysis	103	4	**<0.001**
		Healthy individuals	106	3	

p-Values <0.05 are marked in bold.

No trends were identified in the HD population, and subjects were considered to be in steady state. However, a positive trend was observed in the HI population for K^+^ and Cl^−^. Within this population, one and two healthy subjects were identified as being cause of the trend, respectively, and were removed from the dataset. The analysis was repeated, with no further trends observed.

Number of included results, BV estimates obtained using the Bayesian method with their 95 % credibility intervals (95 % CrI), estimated Harris-Brown ratio (HBR) and individuality index (II) are displayed in Table 2. Considering that the median was 10.9 and 10.5 samples per subject for the HD and the HI groups, respectively, the estimated HBR (shown in Table 2) should be below 21.3 % and 21.8 % for the studied population to be considered homogeneous [27].

**Table 2:** Biological variation estimates (Bayesian method) by group with their 95 % credibility intervals (95 % CrI): within-subject (CV_I_), between-subject (CV_G_) and analytical variation (CV_A_).

Measurand	Units	Group	Subjects, n	Results, n	CV_I_ (95 % CrI)	CV_G_ (95 % CrI)	CV_A_ (95 % CrI)	HBR	II
K^+^	mmol/L	Hemodialysis	18	390	6.8 (5.1–8.3)	8.4 (6.8–10.1)	3.3 (3.0–3.6)	45.7^a^	0.8
		Healthy individuals	25	527	4.0 (3.4–4.7)	3.1 (2.0–4.6)	3.6 (3.3–3.8)	20.3	1.3
Na^+^	mmol/L	Hemodialysis	18	394	1.3 (1.1–1.4)	1.9 (1.6–2.2)	<0.1	27.0^a^	0.7
		Healthy individuals	26	548	1.0 (0.9–1.1)	0.9 (0.7–1.2)	<0.1	11.5	1.1
Cl^−^	mmol/L	Hemodialysis	18	394	2.1 (1.7–2.5)	1.6 (1.5–1.7)	<0.1	15.2	1.3
		Healthy individuals	24	513	3.8 (3.3–4.2)	1.2 (1.0–1.4)	<0.1	17.5	3.3

^a^CV_I_, estimate potentially non-homogeneous. HBR, Harris-Brown ratio; II, individuality index are also presented. HBR should be below 21.3 % in hemodialysis patients and 21.8 % in healthy individuals for the studied population to be considered homogeneous.

**Figure 2:**
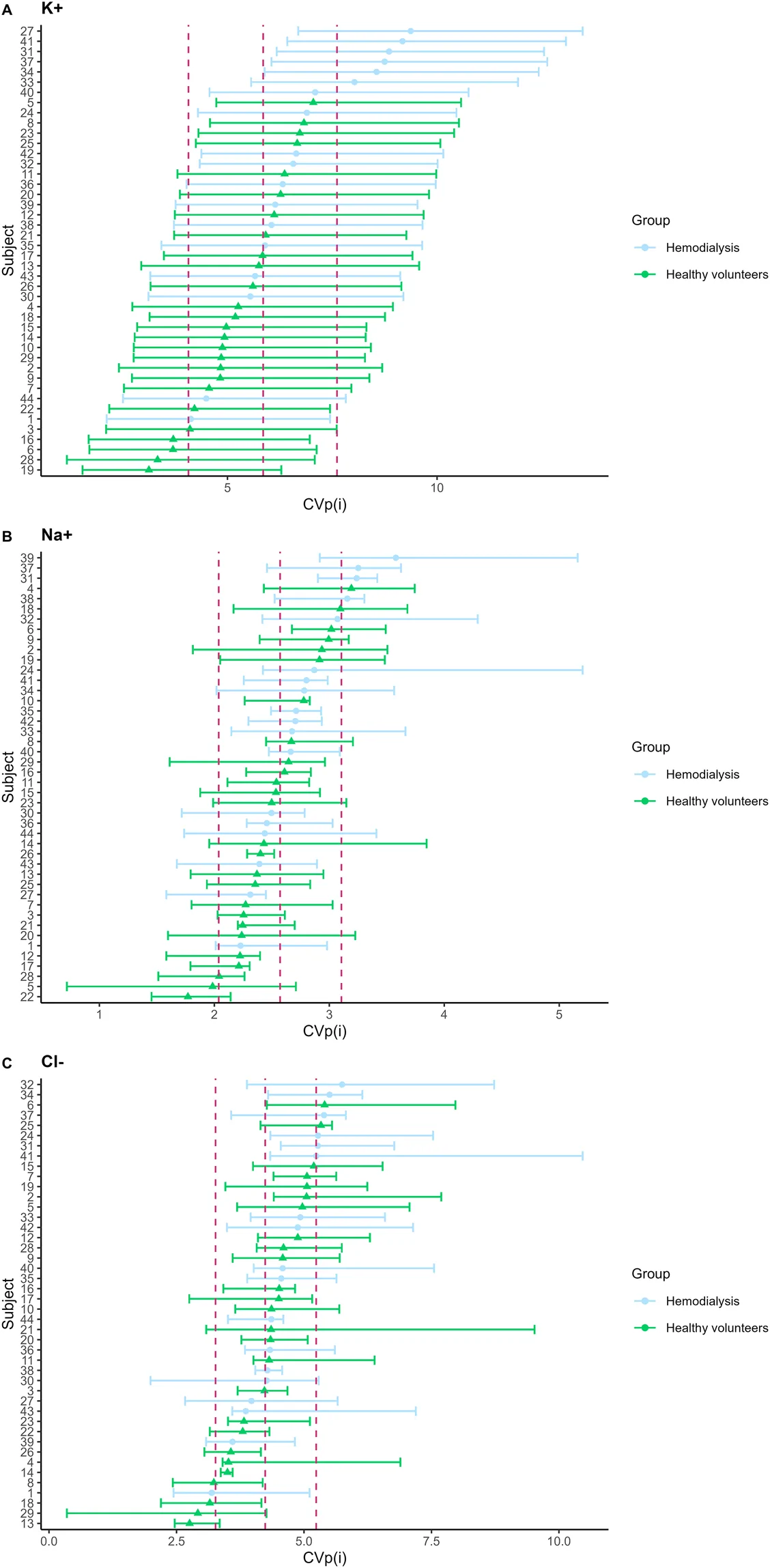
Within-person coefficients of variation (CV_P(I)_) distribution with 95 % credibility intervals. HD subjects are represented in blue and HI participants in green. (A) K^+^, (B) Na^+^, (C) Cl^−^.

In Figure 2, CV_P(I)_ distribution results obtained applying the Bayesian model are shown. Different colours are used to distinguish subgroups: blue lines for HD and green for HI.

Prior to applying ANOVA, analysis of between replicates, samples and individuals outliers was performed. One HD subject was identified as an outlier for Cl^−^ and removed from the dataset. All the information related to the ANOVA calculations is shown in Table 3: number of included subjects and results, percentage of outliers, BV estimates with their 95 % confidence intervals (CI) and II.

**Table 3:** Number of subjects and results included for the calculations after outliers’ analysis, biological variation estimates (CV-ANOVA method) by group with their 95 % confidence intervals (95 % CI): within-subject (CV_I_), between-subject (CV_G_) and analytical variation (CV_A_).

Measurand	Units	Group	Subjects, n	Results, n	Outliers (n, %)	CV_I_ (95 % CI)	CV_G_ (95 % CI)	CV_A_ (95 % CI)	II
K^+^	mmol/L	Hemodialysis	18	371	19 (4.9 %)	9.6 (8.9–10.6)	11.0 (8.0–16.2)	1.8 (1.6–2.0)	0.9
		Healthy individuals	25	507	20 (3.8 %)	4.5 (4.2–5.0)	2.4 (1.6–3.5)	2.1 (1.9–2.3)	1.9
Na^+^	mmol/L	Hemodialysis	18	360	34 (8.6 %)	1.4 (1.3–1.6)	1.9 (1.4–2.7)	0.1 (0.1–0.1)	0.8
		Healthy individuals	26	543	5 (0.9 %)	0.9 (0.9–1.0)	0.8 (0.6–1.2)	0.2 (0.2–0.2)	1.1
Cl^−^	mmol/L	Hemodialysis	17	370	24 (6.1 %)	1.9 (1.8–2.2)	2.2 (1.6–3.2)	0.2 (0.2–0.2)	0.9
		Healthy individuals	24	476	37 (7.2 %)	1.0 (0.9–1.1)	0.4 (0.0–0.5)	<0.1	2.6

II, individuality index.

## Discussion

In this study, we assessed the BV of electrolytes in whole blood with a parallel study design in two groups: healthy individuals and CKD patients under routine HD treatment. Assessment of the two study population groups shows some differences, the HD group consisted mainly of males (13 men and 5 women), while the distribution in the healthy group was the opposite (20 women and 6 men). Participants in the HD group were substantially older than those in the healthy group, with a median age more than twice that of healthy individuals. These demographic differences should be considered when interpreting and comparing results but reflect the well-established epidemiological characteristics of CKD, particularly in advanced stages, which predominantly affects older men [25]. Given the limited sample size and the absence of sex-related differences in BIVAC-compliant BV estimates for electrolytes reported in the literature [31], 32], no stratified analyses by sex were undertaken. However, the influence of age has been scarcely studied.

Statistically significant differences were observed when comparing the concentrations of the three electrolytes studied in the two groups. K^+^ median concentration was higher in the HD group, while Na^+^ and Cl^−^ concentrations were lower. The kidney is the main organ responsible for regulating K^+^, and its loss of function means that K^+^ cannot be eliminated properly due to altered mechanisms such as reduced distal tubular secretion. Additionally, metabolic acidosis further contributes to K^+^ retention, ultimately predisposing patients with CKD to hyperkalemia [33]. As shown in Figure 1, for most patients in the HD group all or almost all their K^+^ results were above the higher RI limit, with only three subjects having concentrations within the RI at all their visits. This is consistent with the relevance and value that the use of personalised RIs would bring to the interpretation of results. The observation of a trend of lower Cl^−^ and Na^+^ concentrations in the HD group may be explained by the compromised capacity to dilute or concentrate urine and tubular damage in CKD patients [33], 34]. In the context of hemodialysis, intermittent correction of acidosis using bicarbonate-rich dialysates temporarily normalises pH but it does not eliminate the underlying tendency towards dilutional hyponatraemia and hypochloremia between HD sessions, especially in patients with fluid overload or dietary sodium restrictions.

Given the large proportion of results excluded prior to ANOVA analysis for all measurands except for Na^+^ in the HI group, the ANOVA-derived estimates should be interpreted with caution and may not be fully representative of the whole population. To put this into perspective and compare it with a methodologically compliant study, in the EuBIVAS [31] dataset, the range of outliers removed before calculating estimates was 0.0–5.6 %. In contrast, the Bayesian approach, which incorporates all observations and is less sensitive to extreme values, may be more appropriate for estimating BV and deriving metrics such as the RCV in datasets with greater heterogeneity. Comparison between results from the ANOVA and Bayesian methods, though uncertainty measures are different, indicate there are differences in CV_I_ estimates observed for those settings where the largest number of results were excluded prior to CV-ANOVA analysis, i.e. K^+^ in HD patients and Cl^−^ in the HI groups.

The HBR is a measure of dispersion of the expected variation within the population and is included in the Bayesian model, allowing for assessing the heterogeneity of data and if a common or average estimate is appropriate for use in the general population. The estimated HBR for Na^+^ and K^+^ for the HD group was higher than that of the HI group, while the opposite was the case for Cl^−^. The estimated HBR should in our study be below 21.3 % and 21.8 % for the HD and HI groups, respectively, to be considered homogeneous. In HD, Na^+^ and K^+^ have HBRs well above 21.3 % (27.0 % and 45.7 %, respectively). Due to the nonhomogeneous CV_I_ distribution, a mean CV_I_ estimate for those measurands should not be considered and the median CV_I_ (50th percentile) would be more correct to apply instead. For Cl^−^ the HBR was below the estimated limit, so we could only consider the population to be homogeneous for the latter. Meanwhile, in HI, all the HBR values were less than 21.8 %. In Figure 2, which shows the CV_P(I)_ per measurand and group, the dispersion of the population can be visually observed.

To assess differences in estimates between subgroups, the lack of overlap between credibility intervals (95 % CrI) may be used. Based on this, there are significant differences between the HI and HD groups for all BV estimates when estimated by the Bayesian method. Except for CV_I_ for Cl^−^, BV estimates were higher in the HD group. The higher CV_I_ in the HD group can reflect that these individuals could have greater lability in their homeostatic regulation mechanisms and may be prone to larger and more abrupt changes in the concentrations of the different electrolytes, since their regulation is conditioned by dialysis treatment.

There is very little literature on BV in CKD and there are no previous BV data for electrolytes in whole blood. Comparing the BV estimates we derived for healthy individuals with global meta-analysis derived BV estimates published in the EFLM BV database [7] in serum, it can be seen that, for the three electrolytes, CV_G_ 95 % CIs overlap, while CV_I_s are significantly different for Na^+^ and Cl^−^. Overall, our population shows a behaviour comparable to that reported for serum/plasma in literature, while a higher degree of within-subject BV is observed within the healthy population in our study. Our BV estimates for HD were also higher, both for CV_I_ and CV_G_, than those in the EFLM database [7]. The estimates have also been compared with two publications that studied electrolytes’ BV in renal patients [35], 36]. Hölzel et al. [35] conducted a study with patients with glomerulonephritis and pyelonephritis to deliver intra-individual BV estimates that were, for the three ions studied, lower than those in our study. Additionally, Fraser and Williams [36] performed a short-term study in patients with impaired renal function where CV_I_ for K^+^ was lower than the CV_I_ in our HD group, but Na^+^ and Cl^−^ ones were higher. All the mentioned results refer to serum/plasma measurements and sampling intervals were different from ours. Moreover, the two BV studies on kidney disease report some methodological limitations, suggesting that the estimates should be interpreted with caution.

RCV allows us to assess whether analytical and within-subject BV may explain the difference between two consecutive results. In the case of high individuality measurands (II<0.6), the RCV is a preferable tool to RIs for interpreting consecutive results. When the II is greater than 1.4 (low individuality), population-based RIs may be used [28]. Considering the II data (Tables 2 and 3), the HD group showed higher individuality (lower II). No II is below the 0.6 threshold, however, most of them were lower than 1.4 for both groups, except for Cl^−^ in HI. Apart from Cl^−^ in HI, all the studied measurands showed medium to high individuality, suggesting that, at least the studied populations might benefit from the use of prRIs to interpret serial results of blood gas electrolytes (measured in whole blood by direct potentiometry).

To the best of our knowledge, this is the first time that BV estimates for electrolytes in whole blood have been calculated and differences between healthy individuals and hemodialysis patients assessed. Furthermore, this is the only study that is fully BIVAC [6] compliant and the results here presented could be used as a baseline and hyperparameters in further studies. It is also important to note that the BV estimates presented in this study are specific to our methodology (direct potentiometry, ABL90 blood gas analyser) and sampling interval (weekly samples).

The relevance of these results extends beyond statistical comparison. In clinical practice, hemodialysis patients undergo frequent laboratory monitoring, so having disease-specific BV and RCV estimates may enable more precise interpretation of serial results, potentially improving therapeutic adjustments, early detection of instability, and overall patient outcomes. Thus, future studies on the use of these data for real-life application in CKD patient care are warranted.

In conclusion, the results obtained confirm significant differences in the BV of total blood electrolytes between healthy individuals and patients undergoing hemodialysis. These findings point to the need for disease-specific reference tools and open new opportunities for personalized laboratory medicine in HD monitoring. Additionally, CV_I_ estimates in whole blood from healthy individuals appeared to be higher than those reported for serum/plasma.

### Limitations

Although the project has achieved its main objective, it is important to acknowledge limitations. One limitation is that the sample size, while robust for this type of study, did not reach the original recruitment target (25 subjects for HD and 40 for HI). Additionally, the demographic differences could represent a bias when comparing results, especially in terms of age, where further studies would be of interest. Sample collection for HD was restricted to mid-week sessions to standardise the preanalytical situation; however, this may not fully represent HD patients’ complete physiological fluctuation and further studies would be desirable in order to gain a better understanding of intradialysis physiological characteristics. It is well known that K^+^ concentration is affected by hemolysis, which might represent a preanalytical limitation of this study, as the equipment used does not allow for detection of hemolysis. Although markedly aberrant results have been excluded during outlier detection, mild or moderate hemolysis could not be identified and might have contributed to the observed variability, particularly for K^+^. The lack of BV estimates for the type of sample (whole blood) and one of the included populations (CKD patients) constitutes a limitation for setting priors in the Bayesian method. Because of this, we needed to resort to BV estimates in serum for healthy individuals; therefore, to minimise this lack, a lower weight in the model was applied.
